# A novel *TP63* variant in a patient with ankyloblepharon-ectodermal defect–cleft lip/palate syndrome and Rapp–Hodgkin syndrome-like ectodermal dysplasia

**DOI:** 10.1038/s41439-022-00186-w

**Published:** 2022-05-20

**Authors:** Asuka Hori, Ohsuke Migita, Nobutaka Isogawa, Fumio Takada, Kenichiro Hata

**Affiliations:** 1 Department of Medical Genetics and Genomics, Kitasato University Graduate School of Medical Sciences, Kanagawa, Japan; 2 Department of Genetics and Genomics, Kitasato University Hospital, Kanagawa, Japan; 3 Department of Maternal-Fetal Biology, Research Institute, National Center for Child Health and Development, Tokyo, Japan; 4 Faculty of Medicine, University of Tsukuba, Ibaraki, Japan; 5 Division of Pedodontics/Orthodontics, Department of Surgical Specialties, National Center for Child Health and Development, Tokyo, Japan

**Keywords:** Genetic testing, Disease genetics

## Abstract

Ankyloblepharon-ectodermal defect–cleft lip/palate syndrome and Rapp–Hodgkin syndrome are well-known *TP63*-related autosomal-dominant genetic disorders with various similar ectodermal dysplasias. In this study, whole-exome sequencing revealed a novel, potentially pathogenic *TP63* nonsense variant (NM_001114980.2:c.25 C > T: p.Gln9Ter) in a patient with an atypical clinical phenotype. This variant was detected near translation initiation sites and has an effect only on ΔNp63α, the short isoform protein product of the *TP63* gene.

*TP63*-related disorders include various combinations of limb anomalies, ectodermal dysplasias, and orofacial clefts. Herein, we report a case of ankyloblepharon-ectodermal defect–cleft lip/palate (AEC) syndrome and Rapp–Hodgkin syndrome (RHS)-like ectodermal dysplasia. The patient was a 12-year-old male and the second child (Fig. 1a, III-4) of nonconsanguineous, healthy Japanese parents (Fig. 1a, II-1 and II-6). His parents and elder sister (Fig. 1a, III-3) had no relevant medical histories. The patient was delivered at 39 weeks gestation without any obstetric complications. He weighed 3,116 g and had an Apgar score of 8/9. At birth, bilateral cleft lip and palate and partial scalp defects were recorded. The patient was accordingly referred to the Department of Otorhinolaryngology at Kitasato University. Neonatal auditory brainstem response analysis showed bilateral elevated hearing thresholds >60 dB. Cleft lip surgery was performed at 3 months of age, and palate repair was performed at 13 months of age. When the patient was 1-year-old, he was diagnosed with ectrodactyly ectodermal dysplasia-cleft lip/palate (EEC) syndrome (OMIM#604292), a *TP63*-related disorder without limb malformations, based on his cleft lip and palate and ectodermal dysplasia. Recurrent otitis media was recorded at 2 years of age, and tympanostomy tube insertion was performed in both ears at 4 years of age. Scalp dermatitis and folliculitis were also observed. The patient’s skin and hair were light-colored, hypoplasia of the nails was observed, and the pain reaction was weak. Abnormal perspiration, keratosis, and dental dysplasia were not noted. At 4 years and 4 months of age, his verbal intelligence quotient (IQ) was 79, performance IQ was 123, and full-scale IQ was 100 as per the *Wechsler Preschool and Primary Scale of Intelligence*. The patient was referred to the community rehabilitation center for speech–language rehabilitation. At 7 years of age, secondary bone grafting of the alveolar cleft was performed, and the oronasal fistula was closed. Rehabilitation was conducted until 8 years of age. The patient demonstrated signs of skeletal crossbite associated with midfacial hypoplasia for which future orthopedic surgery seemed to be necessary. The tympanostomy tubes were removed when he was 13 years old, and he was subsequently diagnosed with conductive hearing loss. His hearing levels were 35 dB on the right and 23.8 dB on the left. Speech developmental problems were not evident.

**Fig. 1:**
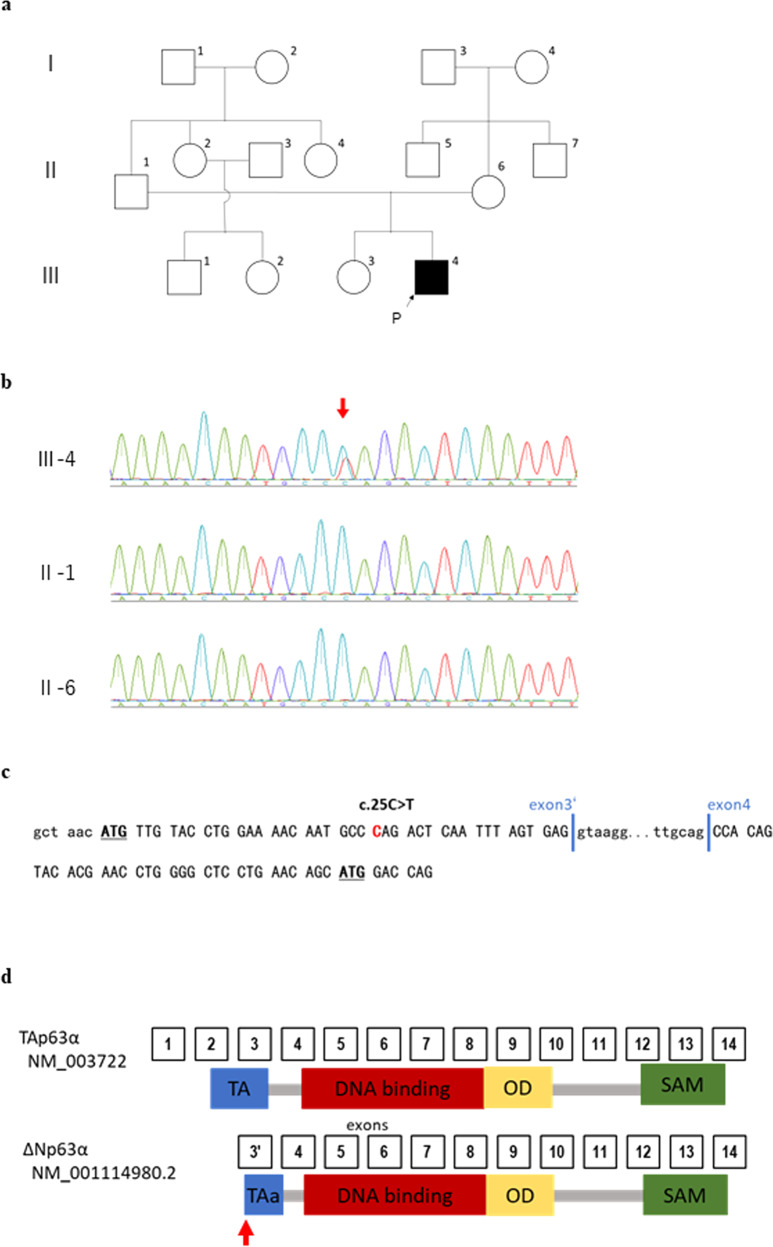
Patient information and the nonsense *TP63* variant. **a** Family pedigree. **b** Sequencing confirmation of the *TP63* variant. The red arrow indicates the position of the variant. **c** The translated sequence of ΔNp63α (NM_001114980.2). **d** Quoted and modified from Ref. ^7^. TP63 isoforms. TA transactivation, OD oligomerization domain, SAM sterile alpha motif (NM_003722, NP_003713.3, NM_001114980.2, NP_001108452.1). The red arrow indicates the position of the variant in this patient.

Detailed genetic analyses were conducted as per the protocol approved by the Ethics Committee of the National Center for Child Health and Development and the Kitasato University Hospital. Written informed consent was obtained from the patient and his parents. Whole-exome sequencing (WES) was performed as described in our previous report^1^. The minor variant frequencies in the general population were estimated using the 1000 Genomes Project database (http://www.internationalgenome.org), Human Genome Variation Database (http://www.hgvd.genome.med.kyoto-u.ac.jp), and Japanese Multi Omics Reference Panel (https://jmorp.megabank.tohoku.ac.jp). WES and Sanger sequencing revealed a heterozygous de novo variant in *TP63* (NM_001114980.2:c.25 C > T: p.Gln9Ter) (Fig. 1b). This variant was not registered as a pathogenic variant in the Human Genome Mutation Database (http://www.hgmd.cf.ac.uk/) or ClinVar (https://www.ncbi.nlm.nih.gov/clinvar/).

*TP63* is a transcription factor gene. Heterozygous pathogenic variants in *TP63* cause multiple syndromes, including EEC syndrome, AEC syndrome (OMIM #106260), RHS (OMIM #129400), acro-dermato-ungual-lacrimal-tooth (ADULT) syndrome (OMIM #103285), and split hand/foot malformation (OMIM #605289), depending on the combinations of ectodermal dysplasia, limb malformation, and cleft lip/palate symptoms^2^. Since *TP63* is pleiotropic, different variants could lead to various disorders, and several syndromes consequently have variant hotspots on *TP63*. EEC syndrome, which is the most common *TP63*-related disorder, is associated with missense variants in the DNA-binding domain. Alternatively, most patients with AEC syndrome have missense variants in the sterile alpha motif (SAM) domain^2–5^.

The patient in this report had a cleft lip and palate, ectodermal dysplasia, and partial scalp defects but no limb malformation and no signs of ankyloblepharon. Although ankyloblepharon is absent, cleft lip and palate, ectodermal dysplasia, and especially partial scalp loss are characteristic phenotypes of AEC syndrome^6^. On the other hand, the symptoms meet all the clinical features of RHS except anhidrotic ectodermal dysplasia. The neighboring variants of p.Gln9Ter are summarized in Table 1. Three variants, two nonsense and one frameshift, have been reported (c.26delA [p.Q9fsTer23], c.31 C > T [p.Q11Ter], and c.46 C > T [p.Q16Ter]) in patients with AEC/RHS-like ectodermal dysplasia^7,8^. *TP63* encodes multiple isoforms^3,9,10^, and two major isoforms produce proteins called TAp63α and ΔNp63α, which share exons 4–14 (Fig. 1c, d). The previously reported p.Q9fsTer23 and p.Q11Ter variants produce N-terminal truncated mutants of the short isoform protein ΔNp63α but do not affect the long isoform protein TAp63α^7^. The p.Gln9Ter variant most likely shows molecular pathogeneses similar to those described above^7^. Nonsense-mediated mRNA decay degrades mutated mRNA with variants that introduce premature termination codons (PTCs). However, when a PTC occurs close to an endogenous transcription start site (TSS), the N-terminus truncated protein could be produced using a neighboring downstream ATG as an alternative TSS. A previous report strongly suggested that the p.Q11Ter, p.Q9fsTer23, and p.Q16Ter retranslations started from the ATG next to the PTC and expressed the truncated ΔNp63α^7^. This suggests that intact TAp63α and truncated ΔNp63α are expressed in the present patient. A patient carrying a missense variant, p.N6H, was clinically diagnosed with ADULT syndrome^11^, which has clinical features that are distinct from those of AEC syndrome. This may be because the p.N6H missense variant produces mutated full-length TAp63α and ΔNp63α.

**Table 1:** Summary of the clinical results of reported *TP63*-related disorders with variants in the vicinity of the variant in the present case.

Variant and clinical diagnosis	p.N6H ADULT (ref. ^11^)	p.Q9Ter^a^ AEC/RHS	p.Q9fsTer23 AEC/RHS (ref. ^7^)	p.Q11Ter AEC/RHS (ref. ^7^)	p.Q16Ter AEC/RHS (ref. ^7^)	Typical AEC^b^
Ankyloblepharon	NA	−	+	−	+	+
Skin trauma	−	+	NA	−	NA	+
Nail abnormalities	+	+	−	1/2^c^	−	+
Dental dysplasia	+	−	−	1/2^c^	−	+
Abnormal sweating	NA	−	−	+	−	+
Cleft lip	NA	+	−	1/2^c^	+	±
Cleft palate	NA	+	+	1/2^c^	+	+
Deformities of the extremities	+	−	−	−	−	±

±Both presence and absence are reported.

*ADULT* acro-dermato-ungual-lacrimal-tooth syndrome, *AEC* ankyloblepharon-ectodermal defects-cleft lip/palate syndrome, *NA* not available, *SAM* Sterile alpha motif.

^a^Present case.

^b^AEC patients with variants in the SAM domain.

^c^Findings in one of the two affected individuals in the family.

We observed that this patient had milder skin manifestations than typical AEC syndrome patients. Most variants in patients with AEC syndrome with severe skin manifestations have been reported in the SAM and transactivation-inhibitory domains in the C-terminus of full-length TAp63α and ΔNp63α^5^. In contrast, we speculate that the skin symptoms in the present patient were milder because there was no variant in the C-terminus, and the long isoform protein was intact. Owing to variations in the severity of ectodermal dysplasia in *TP63-*related disorders^12^, genetic tests will be helpful in making a definitive diagnosis.

In conclusion, we identified a novel nonsense *TP63* variant in a short-isoform-specific exon that may cause AEC/RHS-like ectodermal dysplasia. *TP63*-related disorders exhibit diverse phenotypes. It is believed that some atypical *TP63*-related disorders, such as that in the present patient, might go undiagnosed. We also believe that additional cases can be identified through genetic testing to contribute to a better understanding and improved medical management of *TP63-*related disorders.

## HGV database

The relevant data from this Data Report are hosted at the Human Genome Variation Database at 10.6084/m9.figshare.hgv.3119.
